# From Data Integration to Precision Medicine: A Value-Based Healthcare Approach for Sarcoma Care

**DOI:** 10.3390/jcm13216500

**Published:** 2024-10-30

**Authors:** Bruno Fuchs, Philip Heesen

**Affiliations:** 1 Sarcoma Center/IPU, Department of Orthopaedics and Trauma, LUKS University Hospital, 6000 Luzern, Switzerland; 2 Faculty of Health Sciences and Medicine, University of Lucerne, Frohburgstrasse 3, 6002 Luzern, Switzerland; 3 Sarkomzentrum KSW, Klinik für Orthopädie und Traumatologie, Kantonsspital Winterthur, 8400 Winterthur, Switzerland; 4 Medical Faculty, University of Zurich, 8032 Zurich, Switzerland; philip.heesen@uzh.ch

**Keywords:** value-based healthcare and sarcoma, data harmonization, interoperability, digital transformation, ontologies and standardized coding systems, predictive analytics

## Abstract

The transformation of healthcare from a fee-for-service model to value-based care is particularly crucial in managing complex and rare diseases like sarcoma, where data fragmentation and variability present significant challenges. This manuscript reviews strategies for structured and harmonized data integration—a critical precursor to precision medicine in sarcoma care. We demonstrate how standardizing data formats, ontologies, and coding systems enable seamless integration of clinical, economic, and patient-reported outcomes across institutions, paving the way for comprehensive predictive analytics. By establishing robust value-based healthcare (VBHC) frameworks through digital transformation and predictive models, including digital twins, we create the foundation for personalized sarcoma treatment and real-world-time clinical decision-making. The manuscript also addresses practical challenges, including the need for system standardization, overcoming regulatory and privacy concerns, and managing high costs. We propose actionable strategies to overcome these barriers and discuss the role of advanced analytics and future research directions that further enhance VBHC and precision medicine. This work outlines the necessary steps to build a cohesive, data-driven approach that supports the transition to precision medicine, fundamentally improving outcomes for sarcoma patients.

## 1. Introduction

### 1.1. Background and Context

Sarcomas represent a rare and heterogeneous group of malignancies, originating from mesenchymal tissues such as bones, muscles, fat, and connective tissues [1]. Accounting for only about 1% of all adult cancers and approximately 15% of pediatric cancers, sarcomas pose unique challenges in diagnosis, treatment, and research [2]. The complexity of sarcomas is exacerbated by the fact that there are over 70 distinct subtypes, each with its own histological and genetic characteristics [3]. This diversity contributes to fragmented care and a lack of standardized treatment protocols, as the rarity of each subtype often limits the availability of comprehensive data and evidence-based guidelines [4,5].

The fragmentation in sarcoma care is further compounded by the disparate sources of data collected across different institutions and countries. These data sources include clinical records, patient-reported outcomes, imaging studies, genomic data, and economic metrics, all of which are often siloed within specific registries or institutions [6]. The lack of integration among these diverse datasets results in missed opportunities for comprehensive analysis and hinders the development of holistic treatment approaches [7]. Moreover, the current reliance on retrospective and often incomplete data, which may not fully capture the nuances of individual patient journeys, further limits the ability to deliver personalized and effective care [8].

### 1.2. Purpose of This Review

This paper seeks to address these challenges by highlighting the critical need for structured and harmonized data integration as a foundational step towards transitioning from a volume-based to a value-based healthcare (VBHC) model in sarcoma care [9]. The aim is to establish a unified framework that allows for the seamless integration of clinical, economic, and patient-reported data, enabling more informed decision-making and thereby improving patient outcomes and decreasing healthcare spending. By building this foundation, the paper also sets the stage for future advancements in precision medicine, where the insights gained from integrated data can be leveraged to develop personalized treatment strategies that are both effective and cost-efficient [9].

### 1.3. Fragmented Care and Data Approach

The current landscape of sarcoma care is characterized by significant fragmentation, both in terms of care delivery and data management [10,11]. The rarity of sarcomas often necessitates that patients receive care from multiple specialists across different institutions, leading to inconsistencies in treatment approaches and gaps in care coordination [12]. This fragmentation is mirrored in the data management practices within the field, where disparate sources of data are collected and stored in isolation, preventing a comprehensive understanding of patient outcomes [13].

This fragmented approach to data management has significant consequences for clinical research and ultimately treatment outcomes. Without a unified framework for data integration, clinicians are unable to access the full spectrum of information needed to make informed decisions [10,14]. This lack of comprehensive data also impedes the ability to conduct robust clinical research, as the fragmented datasets do not provide a complete picture of patient experiences and outcomes [15]. Moving towards a prospective data assessment model, incorporating real-world-time data collection and integration across all dimensions of sarcoma care is essential [8]. This shift will enable the development of standardized treatment protocols and facilitate the implementation of VBHC principles, ultimately leading to better care coordination and improved patient outcomes.

### 1.4. The Importance of VBHC

Traditional volume-based care models, which incentivize the provision of more services regardless of their impact on patient outcomes, are increasingly being recognized as unsustainable, particularly in resource-intensive fields such as oncology [16]. In contrast, VBHC emphasizes the importance of achieving the best possible patient outcomes relative to the costs incurred, making it a more sustainable and patient-centered approach to care delivery [17,18].

In sarcoma care, where the costs of treatment can be exceptionally high and the availability of evidence-based guidelines is limited, VBHC offers a framework for optimizing resource allocation and ensuring that care decisions are driven by the goal of maximizing patient value [19,20]. By focusing on quality and outcomes, VBHC encourages the development of more effective and efficient treatment strategies that are tailored to the individual needs of each patient. This approach not only reduces inefficiencies within the healthcare system but also enhances the overall quality of care, leading to better patient outcomes and higher levels of patient satisfaction.

## 2. Materials and Methods

### 2.1. Interoperable Digital Platform

The SSN-Sarconnector platform serves as an exemplary model for demonstrating the importance of interoperability in integrating diverse global registries and data sources [6,21]. While the discussion uses Sarconnector as a reference point, the focus remains on the underlying principles that make such platforms effective, emphasizing the need for a universally accessible and interoperable system [8]. This approach allows for the harmonization of structured data from various healthcare systems worldwide, supporting a more comprehensive understanding of patient outcomes, cost-effectiveness, and overall care quality—key components for advancing the VBHC model in sarcoma care [22]. The Sarconnector captures four data dimensions in a fully standardized way, enabling harmonization with other datasets.

### 2.2. Four Data Dimensions

1. Clinician-Reported Outcome Measures (CROMS):

The structured integration of clinical data through Clinician-Reported Outcome Measures (CROMS) is essential for creating a comprehensive view of patient care [6]. CROMS focus on clinical metrics and outcomes, such as treatment efficacy, safety, and overall patient management, providing insights into the effectiveness of various diagnostic and therapeutic approaches across different sarcoma subtypes. Harmonizing these clinical data points ensures that care is consistently measured and evaluated, fostering improvements in treatment protocols and outcomes.

2. Patient-Reported Outcome Measures (PROMS):

Incorporating Patient-Reported Outcome Measures (PROMS) into the data integration framework provides a holistic view of patient wellbeing, extending beyond clinical metrics to include the patient’s perspective on their health and quality of life [6]. PROMS capture patient feedback on their symptoms, physical functioning, and emotional wellbeing, which are vital for understanding the impact of sarcoma treatments from the patient’s perspective. This dimension reinforces VBHC’s emphasis on patient-centered care by ensuring that the patient’s voice is integral to the overall assessment of care quality.

3. Economics-Reported Outcome Measures (ECOMS):

Economic data integration through Economics-Reported Outcome Measures (ECOMS) is crucial for developing the Sarcoma Care Cost Score (SCCS), detailed below [6]. This score provides a standardized metric for evaluating the cost-effectiveness of different treatments, facilitating more informed decision-making and better resource allocation within the VBHC framework. Integrating cost data from multiple sources ensures that the SCCS is comprehensive and reflective of the real-world costs associated with sarcoma treatment.

4. Patient-Centric Omics Measures (PCOMS):

Patient-Centric Omics Measures (PCOMS) focus on integrating molecular and genomic data, providing a personalized understanding of each patient’s disease profile [6]. PCOMS enable the identification of specific biomarkers and genetic alterations that can influence diagnosis, treatment response, and prognosis. This dimension is crucial for advancing precision medicine, as it allows for the tailoring of therapies based on individual molecular characteristics, leading to more effective and targeted treatment strategies.

### 2.3. The Role of AI in Data Structuring

While AI holds significant promise in transforming unstructured data into structured formats, the process is inherently limited by the quality and completeness of the original data input [23,24]. AI can extract patterns and insights from unstructured data, such as free-text clinical notes, but if critical information is absent or inconsistently recorded, the structured output will reflect these deficiencies [25]. This limitation underscores the importance of structured and standardized data entry at the point of care, where clinical, economic, patient-reported, and molecular data must be systematically captured. By ensuring that data are structured from the outset, healthcare systems can minimize post-processing errors, enhance the efficiency and accuracy of AI-driven analyses, and support the development of robust, interoperable data systems critical for VBHC and future precision medicine applications [26,27,28,29]. A minimal dataset incorporated into clinical practice can ensure that critical information is fully captured [8].

### 2.4. Real-World-Time Data Integration

Integrating both real-world and real-time data is critical for enhancing the relevance and timeliness of clinical decision-making [8]. Real-world data provide insights based on actual patient experiences in diverse settings, while real-time data enable immediate feedback and adjustments in care. Together, these data types ensure that the platform supports dynamic and responsive care strategies tailored to the evolving needs of sarcoma patients. This integration is vital for advancing VBHC and laying the groundwork for future developments in precision medicine.

### 2.5. Harmonization and Standardization

Data Harmonization:

Harmonizing diverse data types across different healthcare systems is essential for creating an interoperable digital platform [28,30]. Achieving data harmonization involves utilizing standardized data formats, ontologies, and coding systems that enable consistent interpretation and integration of data from multiple sources [31]. For example, standardized clinical terminologies such as SNOMED CT (Systematized Nomenclature of Medicine—Clinical Terms) provide a comprehensive, multilingual clinical healthcare terminology that enables uniform representation of clinical content in electronic health records [32,33]. Disease classifications using ICD-10 (International Classification of Diseases, 10th Revision) and ICD-O (International Classification of Diseases for Oncology) ensure consistent coding of diagnoses and tumor characteristics [33], although these may not sufficiently mirror the sarcoma entities in particular. Laboratory tests and results can be standardized using LOINC (Logical Observation Identifiers Names and Codes), facilitating the comparability of lab data across institutions [34]. For data exchange protocols, the adoption of HL7 FHIR (Fast Healthcare Interoperability Resources) standards is crucial. FHIR defines how healthcare information can be exchanged between different computer systems regardless of how it is stored, allowing for seamless interoperability [34]. Additionally, implementing Common Data Models (CDMs) like the OMOP CDM (Observational Medical Outcomes Partnership Common Data Model) allows disparate data sources to be transformed into a consistent format, enabling large-scale analytics and research [35].

To ensure the quality of data harmonization, specific measurements and indicators are essential [36]. These include the following: Data Completeness: ensuring all required data fields are captured across datasets. Data Accuracy: verifying that the data correctly represents real-world scenarios, which can involve cross-referencing with source documents or validation studies. Data Consistency: maintaining uniform data formats and units across datasets to prevent discrepancies during integration. Data Timeliness: updating data promptly to reflect the most current information, which is critical for real-world-time data integration.

Implementing a Data Quality Assessment Framework can help regularly evaluate these metrics. For instance, the framework may include automated data validation rules, routine data quality reports, and processes for correcting identified errors. Harmonization efforts are critical for overcoming variability in data collection practices and ensuring that all data contribute meaningfully to the overall assessment of care quality [37]. By incorporating these standardized terminologies, coding systems, data models, and quality measurements, robust data harmonization frameworks facilitate effective integration and analysis of information from global networks, ultimately advancing VBHC in sarcoma care.

Standardization of Metrics:

To measure and improve care quality globally, developing standardized metrics that can be applied consistently across different healthcare settings is essential [38,39,40,41,42]. This section will discuss creating standardized metrics, exemplified using the Sarcoma Clinical Quality Score (SCQS) and the Sarcoma Care Cost Score (SCCS). These metrics provide a common language for evaluating care quality and cost-effectiveness, enabling meaningful comparisons and benchmarking across institutions. Standardization efforts also support transitioning to a VBHC model by ensuring that care is consistently measured and optimized based on universally accepted criteria.

### 2.6. Practical Challenges in Implementation

Implementing structured and harmonized data integration across diverse healthcare systems presents several practical challenges that must be addressed to realize the full potential of Value-Based Healthcare (VBHC) and precision medicine in sarcoma care.

#### 2.6.1. Standardization Across Systems

Healthcare systems worldwide utilize a variety of electronic health record (EHR) systems with differing data formats, terminologies, and standards, making data interoperability a significant challenge [37,43,44]. Aligning these disparate systems requires adopting common data standards and terminologies, which can be complex and resource-intensive [45]. Efforts to standardize data entry and coding practices must consider variability in clinical workflows and the need for extensive training of healthcare professionals.

#### 2.6.2. Regulatory Hurdles

Data sharing and integration must comply with various regulatory frameworks governing patient privacy and data protection, such as the General Data Protection Regulation (GDPR) in Europe and the Health Insurance Portability and Accountability Act (HIPAA) in the United States [46]. Navigating these regulations can be challenging, especially when collaborating across international borders. Ensuring compliance requires careful planning, legal expertise, and robust data governance policies.

#### 2.6.3. Privacy and Data Security Concerns

Protecting patient privacy and ensuring data security are paramount when integrating sensitive health information [47]. Concerns about data breaches and unauthorized access can hinder data-sharing initiatives. Implementing strong security measures—such as encryption, access controls, and regular security assessments—is essential. Additionally, obtaining informed consent from patients and maintaining transparency about data usage can help build trust and facilitate data sharing.

#### 2.6.4. Limitations of Technologies and Solutions

High Costs

Implementing advanced technologies for structured and harmonized data integration can involve significant financial investment. The costs associated with purchasing hardware and software, upgrading existing systems, and training staff may be prohibitive for some institutions, particularly in resource-limited settings. These expenses can hinder the widespread adoption of such technologies.

Solutions: To mitigate high costs, institutions can explore cost-effective alternatives such as open-source software and cloud-based solutions that reduce upfront investments. Collaborative funding models, partnerships with technology providers, and government grants can also help distribute costs.

Need for Structured Data Input

The effectiveness of data integration relies on the availability of structured data. Transitioning from unstructured to structured data input requires changes in clinical documentation practices, which may increase the workload for healthcare providers and encounter resistance [8].

Solutions: Implementing user-friendly data entry interfaces and integrating structured data-capture seamlessly into existing workflows can minimize disruption. Providing training and support for clinicians is essential to facilitate adoption. Additionally, employing natural language processing (NLP) tools can help extract structured data from unstructured clinical notes, reducing the burden on clinicians.

Potential Accuracy Issues

Technologies like AI and machine learning algorithms are dependent on the quality and quantity of available data. Inaccuracies can arise from biased datasets, insufficient data volume, or errors in data entry, potentially leading to unreliable outputs.

Solutions: Ensuring data quality through validation protocols and using diverse, representative datasets can improve algorithm accuracy. Regularly updating and monitoring algorithms helps maintain their reliability. Involving multidisciplinary teams, including clinicians and data scientists, can enhance oversight and address potential biases or errors.

### 2.7. Use of AI Language Model Assistance

In the preparation of this manuscript, the authors utilized the AI language model ChatGPT (developed by OpenAI, San Francisco, CA, USA; www.chatgpt.com, accessed 20 October 2024) to assist in drafting and refining the text. The AI tool was employed to generate initial drafts, suggest phrasing, and improve the clarity and flow of the manuscript. All AI-generated content was thoroughly reviewed, edited, and validated by the authors to ensure accuracy and adherence to scientific standards.

## 3. Advancing VBHC as a Precursor to Precision Medicine

### 3.1. Establishing VBHC

#### 3.1.1. VBHC as the Foundation

VBHC serves as the essential foundation for the eventual integration of precision medicine in sarcoma care [17,19]. VBHC emphasizes the importance of improving patient outcomes relative to the costs of care, a principle that is particularly critical in rare and complex diseases like sarcoma [16]. Establishing VBHC creates the necessary infrastructure for precision medicine by ensuring that care is consistently measured and optimized based on universally accepted metrics. As healthcare shifts from volume to value, structured data integration becomes a vital tool in laying this foundation [16]. This structured data—comprising clinical, economic, and patient-reported outcomes—serves as the baseline for developing more personalized care strategies, enabling a smoother transition to precision medicine. Furthermore, precision medicine, which optimizes the therapeutic outcome and resource use, enhances VBHC.

#### 3.1.2. Structured Data’s Role

The role of structured data in VBHC cannot be overstated [6,48,49]. It provides the benchmarks needed to guide future precision medicine developments by offering a clear, quantifiable understanding of what constitutes effective care. By integrating real-world-time data, clinicians can not only track patient outcomes but also refine treatment protocols based on real-time insights [7,8]. Real-time insights allow for rapid adoption of new clinical protocols and the efficient evaluation of its success. This integration is pivotal in transitioning from traditional, retrospective care models to a dynamic, data-driven approach that continuously evolves with the needs of patients.

### 3.2. Prospective Data Collection and Its Impact

From Retrospective to Prospective:

The transition from retrospective analysis to a prospective data collection framework marks a significant evolution in sarcoma care [7,50,51]. Retrospective data, while valuable, often reflects past trends that may not fully capture the complexities of current patient populations or the nuances of modern treatments. By moving to prospective data collection, healthcare providers can gather real-world-time data that more accurately reflects current clinical practices and patient responses [44]. This shift allows for the continuous updating of treatment protocols, leading to more timely and effective interventions. Furthermore, prospective data serves as a more robust foundation for establishing evidence-based guidelines, which are critical for the consistent application of VBHC principles.

Impact of Prospective Data on Treatment Protocols:

The impact of prospective data collection on treatment protocols is profound [8,37,43]. By capturing data in real-time, clinicians can make more informed decisions that are directly aligned with the most current evidence. This approach enhances the precision and personalization of care, enabling the development of guidelines that are not only evidence-based but also adaptable to the individual needs of each patient. Prospective data collection thus plays a crucial role in refining treatment strategies and improving overall patient outcomes within the VBHC framework.

### 3.3. Linking VBHC to Future Precision Medicine

Building Blocks:

Structured and harmonized data collected under the VBHC framework serves as the building blocks for future innovations in precision medicine, including AI integration, digital twinning, benefit-harm modeling, and other advanced technologies [52,53]. As these technologies evolve, the groundwork laid by VBHC ensures that their implementation is both effective and equitable, with a clear focus on improving patient outcomes while managing costs.

The Role of VBHC in Future Precision Medicine:

VBHC’s role in facilitating the transition to precision medicine is multifaceted [27]. By establishing a care model that prioritizes outcomes and efficiency, VBHC ensures that new technologies are integrated into clinical practice in a way that maximizes their potential benefits. This includes ensuring that AI-driven insights and digital twin simulations are grounded in real-world-time data and are applicable across diverse patient populations [53]. The VBHC model also promotes equity in the adoption of precision medicine, ensuring that all patients, regardless of their location or socioeconomic status, have access to the latest advancements in care. This equitable approach is essential for realizing the full potential of precision medicine in sarcoma treatment.

### 3.4. Measuring the Impact of Initiatives Through Outcomes and Benchmarks

Evaluating these initiatives is crucial for demonstrating their value in advancing Value-Based Healthcare (VBHC) and preparing for precision medicine. To this end, we propose specific outcomes and benchmarks that reflect improvements in patient care and system efficiency, primarily through the Sarcoma Clinical Quality Score (SCQS) and the Sarcoma Care Cost Score (SCCS) [6].

The SCQS is a composite metric quantifying sarcoma care quality across multiple dimensions of clinical performance. It evaluates adherence to evidence-based protocols, monitors patient survival rates (overall and progression-free), tracks complication and recurrence rates, and incorporates patient-reported outcome measures (PROMs) regarding functional status and quality of life. By aggregating these factors, the SCQS provides a comprehensive assessment of clinical effectiveness, enabling benchmarking across institutions and over time.

The SCCS assesses the economic aspect of sarcoma care by analyzing direct medical costs (surgeries, medications, hospital stays, outpatient services), indirect costs (lost productivity, long-term care needs), and resource utilization efficiency (operating room time, diagnostic procedures). This score helps identify cost-saving opportunities without compromising quality, aligning with VBHC’s goal of maximizing patient value.

Implementing the SCQS and SCCS enables healthcare providers to benchmark performance against national and international standards, identify areas for improvement, track progress over time, and inform decision-making through data-driven insights that refine treatment protocols and resource allocation.

Additional metrics include interoperability scores assessing a system’s ability to exchange and interpret shared data; data quality indicators monitoring completeness, accuracy, consistency, and timeliness; and patient satisfaction scores capturing patient experiences to provide a holistic view of care quality.

By incorporating these outcomes and benchmarks, we establish a clear framework for measuring the effectiveness of structured and harmonized data integration in advancing VBHC. This approach ensures tangible improvements in patient outcomes, care quality, and cost-efficiency, ultimately paving the way for precision medicine in sarcoma care.

## 4. Expanding the Framework Globally

### 4.1. Digital Platforms as Enablers

#### 4.1.1. Global Integration Through Digital Platforms

Digital platforms, such as Sarconnector, serve as critical models for integrating and connecting international centers focused on sarcoma care [14,21]. These platforms are designed to foster global collaboration by providing a centralized, interoperable system that harmonizes data across diverse regions and healthcare systems. By enabling seamless data exchange and communication between institutions, these platforms break down geographical barriers, allowing for the sharing of best practices, research findings, and patient outcomes. This global integration is essential for addressing the challenges posed by rare diseases like sarcoma, where patient populations are dispersed and data fragmentation hinders progress.

Sarconnector, for example, is not just a tool but a blueprint for how such platforms can be developed and deployed on a global scale. It exemplifies how interoperability can be achieved through standardized data inputs and harmonized metrics, ensuring that data collected from different parts of the world is consistent and comparable. This level of integration is crucial for building a global network of sarcoma care centers that can work together to improve patient outcomes and advance research.

#### 4.1.2. Hub-And-Spoke Model

The implementation of the hub-and-spoke model through digital platforms represents a strategic approach to the regional and global coordination of sarcoma care [54]. In this model, specialized centers of excellence (hubs) provide advanced care and expertise, while regional hospitals and clinics (spokes) offer more localized services. Digital platforms facilitate this model by enabling real-time communication, data sharing, and coordinated care across the network.

In Switzerland, for example, this model has been effectively used to connect different regions and hospitals, ensuring that patients have access to the highest levels of care regardless of their location [55]. Through Sarconnector and similar platforms, Swiss sarcoma centers can collaborate on complex cases, share diagnostic and treatment data, and ensure that all patients receive care that is consistent with the latest evidence-based guidelines. This model not only improves the quality of care but also makes it more efficient and accessible, serving as a template for global expansion.

### 4.2. Global Collaboration and Standardization

#### 4.2.1. International Partnerships

Building international partnerships is a cornerstone of expanding the VBHC framework globally. Collaborating with global sarcoma organizations such as the Connective Tissue Oncology Society (CTOS), EURACAN, SELNET, Sarcoma Patients EuroNet (SPAEN), and the European Musculo-Skeletal Oncology Society (EMSOS) is essential for standardizing care practices and sharing expertise [10,56,57,58,59]. These partnerships enable the development of globally recognized guidelines and best practices, ensuring that all patients receive the highest standard of care, regardless of where they are treated.

Through these collaborations, data from different countries can be pooled, providing a richer dataset for research and the development of new treatments with increased chances of good generalizability. Moreover, a diverse dataset allows for the evaluation of heterogeneous treatment effects and subsequently individualized treatment effects. This global pooling of data also facilitates the benchmarking of outcomes and the identification of best practices, which can then be disseminated across the network to improve care quality worldwide.

#### 4.2.2. Personalized Guidelines Development

Leveraging global data to create personalized, evidence-based guidelines marks a significant advancement over traditional evidence-based guidelines, which are often based on expert opinion rather than empirical data [24,60,61]. By moving towards guidelines informed by prospective, real-world-time data, the sarcoma care community can develop more accurate and applicable treatment protocols tailored to individual patient needs.

These personalized guidelines are particularly important in rare diseases like sarcoma, where variability in patient responses to treatment is high [45,62]. By using data collected through interoperable digital platforms, clinicians can develop guidelines that are not only evidence-based but also highly specific to the diverse patient populations they serve. This approach ensures that treatment recommendations are grounded in real-world outcomes, making them more effective and relevant to clinical practice.

The shift towards prospective, real-world-time data also allows for the continuous updating of guidelines as new data become available, ensuring that they remain current and reflective of the latest scientific advancements. This dynamic approach to guideline development is a key component of VBHC and a precursor to the broader adoption of precision medicine in sarcoma care.

#### 4.2.3. Challenges in Global Adoption of VBHC

Implementing Value-Based Healthcare (VBHC) globally faces challenges due to cultural differences, economic disparities, and variations in healthcare systems [16,18,63,64]. Cultural beliefs and practices influence patient expectations and acceptance of VBHC principles, potentially hindering adoption in certain regions. Economic constraints, especially in low-resource settings, can limit the availability of the infrastructure and technology necessary for VBHC implementation. Additionally, differing healthcare policies and organizational structures across countries complicate standardization efforts.

To overcome these challenges, it is essential to tailor VBHC models to local contexts by considering cultural sensitivities and resource availability [18]. Capacity building through training and education can empower local healthcare professionals. Engaging stakeholders from diverse regions promotes inclusive collaboration, ensuring that VBHC initiatives address specific local needs. Leveraging cost-effective technologies and advocating for supportive policies can further facilitate global adoption.

### 4.3. Future Directions Enhancing Value-Based Healthcare

#### 4.3.1. Practical Enhancements to VBHC

Looking ahead, several emerging trends have the potential to further enhance Value-Based Healthcare (VBHC) in sarcoma care. Emphasizing cost efficiency over mere cost reduction is essential, focusing on optimizing health outcomes relative to the costs incurred. The integration of advanced analytics and predictive modeling, including artificial intelligence and machine learning, can support personalized treatment plans and improve clinical decision-making. Incorporating personalized medicine and genomics allows for tailored therapies that increase effectiveness and avoid unnecessary costs. Expanding digital health technologies, such as telemedicine and remote monitoring, can improve patient engagement, access, and care efficiency. Enhancing interoperability and data sharing facilitates comprehensive data integration and collaboration across systems. Patient engagement and shared decision-making empower patients, improving adherence, satisfaction, and outcomes. Lastly, developing value-based payment models aligns financial incentives with VBHC goals, encouraging providers to deliver high-quality, efficient care. By embracing these future directions, the healthcare community can build upon the foundation established through structured and harmonized data integration, further advancing VBHC and paving the way for precision medicine in sarcoma care.

#### 4.3.2. Future Research Directions

Advancing VBHC and precision medicine in sarcoma care presents several opportunities for future research:

*Empirical Evaluation of VBHC Implementation*

Conducting multicenter studies to assess the impact of VBHC frameworks on patient outcomes, cost-efficiency, and care quality in sarcoma treatment. Evaluating the effectiveness of standardized metrics like the Sarcoma Clinical Quality Score (SCQS) and the Sarcoma Care Cost Score (SCCS) across diverse healthcare settings will provide valuable insights into their applicability and utility.

*Addressing Implementation Challenges*

Investigating strategies to overcome practical challenges such as standardization across different systems, regulatory hurdles, and privacy concerns. Research into cost-effective solutions and technological innovations can facilitate structured data input and integration, particularly in resource-limited settings.

*Global Adaptation of VBHC Models*

Studying the adaptation of VBHC principles to different cultural, economic, and healthcare contexts. Exploring how VBHC can be tailored to local needs will enhance its universal applicability. Assessing the effectiveness of capacity-building initiatives and inclusive collaboration can promote global adoption.

*Integration of Advanced Technologies*

Researching the integration of artificial intelligence, machine learning, and digital health technologies into VBHC frameworks. Evaluating the impact of these technologies on data analysis, predictive modeling, and personalized care can enhance clinical decision-making and patient outcomes in sarcoma care.

By pursuing these research directions, the healthcare community can build upon the foundations established in this review, further advancing VBHC and precision medicine in sarcoma care globally.

## 5. Conclusions

This review has highlighted the critical importance of structured and harmonized data integration as the cornerstone for establishing Value-Based Healthcare (VBHC) in sarcoma care [28]. Transitioning from fragmented, retrospective data approaches to a cohesive, prospective data integration framework is a transformative step toward more effective, efficient, and patient-centered care [6,8,21]. By harmonizing clinical, economic, and patient-reported outcome data across global networks, we create a unified foundation that supports the accurate measurement of care quality and costs, as exemplified by metrics like the SCQS and the SCCS [6].

**Figure 1:**
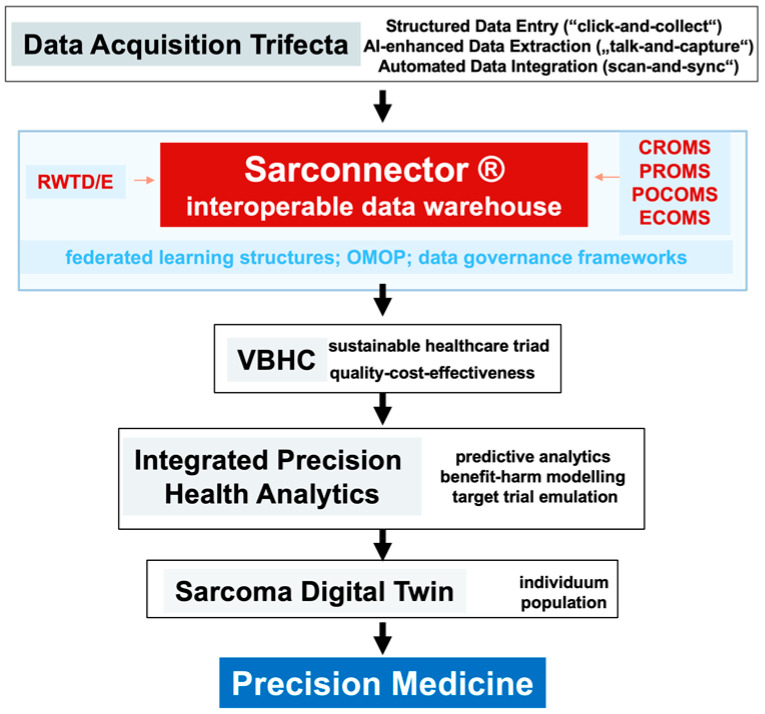
The graphical abstract illustrates the integration of structured and harmonized data into the Sarconnector Interoperable Data Warehouse, which supports value-based healthcare (VBHC) in sarcoma care. Real-world-time data acquisition, including clinician-reported outcomes (CROMS), patient-reported outcomes (PROMS), economic measures (ECOMS), and omics data (PCOMS), feeds into predictive analytics and the Sarcoma Digital Twin. This system facilitates precision medicine by enabling individualized, evidence-based care while optimizing clinical outcomes and cost-effectiveness.

Establishing this VBHC foundation is essential for preparing the ground for the next leap in sarcoma care—precision medicine [9,30]. Robust data systems and metrics enable the integration of advanced technologies such as artificial intelligence (AI), digital twinning, and real-world-time data analytics [52], facilitating the development of personalized treatment guidelines based on evidence rather than tradition. Looking ahead and embracing emerging trends will further enhance VBHC and pave the way for precision medicine. Focusing on cost efficiency, integrating advanced analytics and predictive modeling, expanding digital health technologies, enhancing interoperability, and promoting patient engagement and shared decision-making are vital steps forward. Aligning financial incentives with VBHC goals through value-based payment models will encourage providers to deliver high-quality, efficient care (Figure 1).

The journey toward achieving VBHC in sarcoma care and laying the groundwork for precision medicine requires the concerted effort of global stakeholders. We call upon healthcare providers, researchers, policymakers, and international sarcoma organizations to collaborate in implementing structured and harmonized data integration systems and to embrace emerging innovations that enhance VBHC. This collaboration is essential to ensure that all patients, regardless of their geographic location, have access to high-quality, evidence-based care.

By prioritizing the development of interoperable digital platforms, standardized metrics, and forward-looking strategies, we can pave the way for a new era in sarcoma care—one where treatment decisions are guided by real-world-time data, personalized to each patient, and grounded in the principles of value-based care. This is a feasible, actionable path forward, provided that the global sarcoma community unites in this common goal.

## Data Availability

The data presented in this study are available on request from the corresponding author.
